# *Geomonas oryzae* gen. nov., sp. nov., *Geomonas edaphica* sp. nov., *Geomonas ferrireducens* sp. nov., *Geomonas terrae* sp. nov., Four Ferric-Reducing Bacteria Isolated From Paddy Soil, and Reclassification of Three Species of the Genus *Geobacter* as Members of the Genus *Geomonas* gen. nov.

**DOI:** 10.3389/fmicb.2019.02201

**Published:** 2019-09-25

**Authors:** Zhenxing Xu, Yoko Masuda, Hideomi Itoh, Natsumi Ushijima, Yutaka Shiratori, Keishi Senoo

**Affiliations:** ^1^Department of Applied Biological Chemistry, Graduate School of Agricultural and Life Sciences, The University of Tokyo, Tokyo, Japan; ^2^Bioproduction Research Institute, National Institute of Advanced Industrial Sciences and Technology, Hokkaido, Japan; ^3^Support Section for Education and Research, Graduate School of Dental Medicine, Hokkaido University, Hokkaido, Japan; ^4^Niigata Agricultural Research Institute, Niigata, Japan; ^5^Collaborative Research Institute for Innovative Microbiology, The University of Tokyo, Tokyo, Japan

**Keywords:** *Geomonas*, *Geomonas oryzae* sp. nov., *Geomonas edaphica* sp. nov., *Geomonas ferrireducens* sp. nov., *Geomonas terrae* sp. nov., ferric-reducing bacteria, paddy soil

## Abstract

In paddy soil, bacteria from the family *Geobacteraceae* have been shown to strongly contribute to the biogeochemical cycle. However, no *Geobacteraceae* species with validly published names have been isolated from paddy soil. In this study, we isolated and characterized four novel ferric reducing bacteria in the family *Geobacteraceae* from the paddy soils of three different fields in Japan. The four strains, S43^T^, Red53^T^, S62^T^, and Red111^T^, were Gram-stain negative, strictly anaerobic, chemoheterotrophic, and motile with peritrichous flagella. Phylogenetic studies based on 16S rRNA gene sequences, five concatenated housekeeping genes (*fusA*, *rpoB*, *recA*, *nifD*, and *gyrB*) and 92 concatenated core genes revealed that the four strains belong to the family *Geobacteraceae* and are most closely related to *Geobacter bemidjiensis* Bem^T^ (97.4–98.2%, 16S rRNA gene sequence similarities) and *Geobacter bremensis* Dfr1^T^ (97.1–98.0%). Genomic analysis with average nucleotide identity (ANI) and digital DNA–DNA hybridization (GGDC) calculations clearly distinguished the four isolated strains from other species of the family *Geobacteraceae* and indicated that strains S43^T^, Red53^T^, S62^T^, and Red111^T^ represent independent species, with values below the thresholds for species delineation. Chemotaxonomic characteristics, including major fatty acid and whole cell protein profiles, showed differences among the isolates and their closest relatives, which were consistent with the results of DNA fingerprints and physiological characterization. Additionally, each of the four isolates shared a low 16S rRNA gene sequence similarity (92.4%) and average amino acid identity (AAI) with the type strain of the type species *Geobacter metallireducens*. Overall, strains S43^T^, Red53^T^, S62^T^, and Red111^T^ represent four novel species, which we propose to classify in a novel genus of the family *Geobacteraceae*, and the names *Geomonas oryzae* gen. nov., sp. nov. (type strain S43^T^), *Geomonas edaphica* sp. nov. (type strain Red53^T^), *Geomonas ferrireducens* sp. nov. (type strain S62^T^), and *Geomonas terrae* sp. nov. (type strain Red111^T^) are proposed. Based on phylogenetic and genomic analyses, we also propose the reclassification of *Geobacter bremensis* as *Geomonas bremensis* comb. nov., *Geobacter pelophilus* as *Geomonas pelophila* comb. nov., and *Geobacter bemidjiensis* as *Geomonas bemidjiensis* comb. nov.

## Introduction

The family *Geobacteraceae*, in the order *Desulfuromonadales*, class *Deltaproteobacteria*, was first proposed by Holmes et al. (2004) and currently contains a single genus with a validly published name: *Geobacter* (Garrity et al., 2005; Nevin et al., 2007). The type genus *Geobacter* was first proposed by Lovley et al. (1993) with a description of the type species, *Geobacter metallireducens*. At the time of writing, the family *Geobacteraceae* contained 19 species with validly published names^1^, which were isolated from different environmental sources, e.g., the type species *Geobacter metallireducens* GS-15^T^ was isolated from surficial bottom sediment (Lovley et al., 1993), *Geobacter daltonii* FRC-32^T^ from contaminated sediment (Prakash et al., 2010), *Geobacter toluenoxydans* TMJ1^T^ from tar-oil-contaminated sludge (Kunapuli et al., 2010), and *Geobacter sulfurreducens* PCA^T^ from wastewater biofilm (Caccavo et al., 1994). Besides these isolates, dozens of *Geobacteraceae* clone sequences have been reported from culture-independent analyses in various environments, such as contaminated sediments (Holmes et al., 2009), wastewater (Tejedor-Sanz et al., 2018), paddy soils (Hori et al., 2010; Li et al., 2014), and freshwater lake sediments (Cummings et al., 2003), indicating that the *Geobacteraceae* family is one of the ubiquitous microbial groups in terrestrial and freshwater ecosystems.

Members of the family *Geobacteraceae* were identified as Gram-stain negative, chemoheterotrophic, anaerobic, and capable of reducing Fe(III), Mn(IV), U(VI), elemental sulfur, and humic substances (Childers et al., 2002; Coppi et al., 2007; Prakash et al., 2010; Lovley et al., 2011). Some members were also reported to use other polluting metal ions as electron acceptors, such as Co(III), V(V), Cr(VI), Ag(I), Hg(II), Np(V), and Pu(IV), indicating that *Geobacteraceae* strains have important geochemical effects on natural environments (Lovley et al., 2011; Hernández-Eligio et al., 2018). Other features including extracellular electron transfer, dechlorinating activity, and indirectly contributing to methane production have been well-studied in *Geobacteraceae* (Lovley et al., 2011; Rotaru et al., 2014). Because of these versatile features, *Geobacteraceae* are important microbes in the biodegradation and bioremediation of environmental pollutants and bioenergy production (Shelobolina et al., 2008; Mouser et al., 2009).

Paddy soils, as the largest anthropogenic wetlands on Earth, represent a unique ecosystem that cycles between waterlogged and drained states during long-term paddy cultivation (Kögel-Knabner et al., 2010). Bacterial communities in paddy soils are therefore different from those occurring in other agricultural soils, particularly in terms of the abundance of facultative anaerobic or strictly anaerobic microbes. In our previous metatranscriptomic study of paddy soils in Japan, we found that most identified gene transcripts involved in reductive nitrogen transformation were derived from the order *Deltaproteobacteria*, specifically the genera *Geobacter* and *Anaeromyxobacter* (Masuda et al., 2017). Similar results were also described by Breidenbach et al. (2016), who noted that the genera *Geobacter* and *Anaeromyxobacter*, as potential iron reducers, are notably abundant in the rice rhizosphere environment. However, to date, a *Geobacter* species with a validly published name isolated from paddy soil has not been described. While screening for microorganisms driving nitrogen transformations in paddy soil, four red-colored *Geobacteraceae* strains named as S43^T^, Red53^T^, S62^T^, and Red111^T^ were isolated from three different paddy fields. This study was performed to determine the exact taxonomic status of these four isolated strains by polyphasic characterization.

## Materials and Methods

### Sampling Sites, Enrichment and Isolation

The four microorganisms were isolated from paddy soils collected from different fields in Japan: strains S43^T^ and S62^T^ were isolated from field soil in Nagaoka-shi, Niigata in February 2018, strain Red53^T^ from field soil in Mine-shi, Yamaguchi in March 2018, and strain Red111^T^ from field soil in Kasumigaura-shi, Ibaraki in April 2018. In enrichment experiments for isolating strains S43^T^ and S62^T^, 3 g of fresh paddy soil samples were added aseptically to 50 mL serum bottles containing 30 mL autoclaved modified freshwater medium (MFM) supplemented with 20 mM fumarate and 20 mM acetate as the electron acceptor and donor, respectively (Lovley et al., 1993). The MFM contained the following (in L^–1^): 2.0 g KHCO_3_, 0.02 g MgSO_4_⋅7H_2_O, 0.3 g KH_2_PO_4_, 1.0 g NH_4_Cl, 0.1 g MgCl_2_⋅6H_2_O, 0.08 g CaCl_2_⋅2H_2_O, 0.6 g NaCl, 9.52 g HEPES, 10.0 mL vitamin stock solution (in L^–1^, 2 mg biotin; 2 mg folic acid; 10 mg pyridoxine-HCl; 5 mg thiamine-HCl; 5 mg nicotinic acid; 5 mg aminobenzoic acid; 5 mg Ca-pantothenate; 0.01 mg vitamin B12; 5 mg lipoic acid), and 10.0 mL mineral stock solution (in L^–1^, 12.8 g nitrilotriacetic acid, 1.35 g FeCl_3_⋅6H_2_O, 0.1 g MnCl_2_⋅4H_2_O, 0.024 g CoCl_2_⋅6H_2_O, 0.1 g CaCl_2_⋅2H_2_O, 0.1 g ZnCl_2_, 0.025 g CuCl_2_⋅2H_2_O, 0.01 g H_3_BO_3_, 0.024 g Na_2_MoO_4_⋅2H_2_O, 1 g NaCl, 0.12 g NiCl_2_⋅6H_2_O, 4 mg Na_2_SeO_3_⋅5H_2_O, 4 mg Na_2_WO_4_, pH 6.5). For the isolation of strains Red53^T^ and Red111^T^, such enrichment cultures were performed using soil slurry (air-dried paddy soil/distilled water, 1/1.5) supplemented with only vitamin stock solution, in place of MFM with fumarate and acetate as described above. After the bottle neck was sealed and the air was replaced with N_2_/CO_2_ (80:20, v/v), the enrichment culture bottles were incubated at 30°C without shaking until the soil color changed to gray. Next, under sterile and oxygen-free conditions, the enriched cultures were collected using syringes and serially diluted in oxygen-free water (10^–1^ to 10^–4^), and 100 μL samples of every serial dilution were spread onto R2A agar (Difco, United States) with 20 mM fumarate (modified R2A agar). The plates were then incubated in anaerobic jars equipped with AnaeroPacks (Mitsubishi Gas Chemical, Tokyo, Japan) and oxygen indicators (Mitsubishi Gas Chemical, Tokyo, Japan) at 30°C for 10 days. After repeating this process several times, dozens of red-colored colonies were picked up and purified by subculturing the cells on the same medium. All purified colonies were confirmed using special primers for the family *Geobacteraceae* (Geo564F, Geo840R) as described previously (Cummings et al., 2003). Positive colonies were then selected again with the full 16S rRNA gene sequences and DNA fingerprints, which are described below in the section “Phylogenetic and Genomic Analyses.” Finally, four different red-colored strains, named as S43^T^, S62^T^, Red53^T^, and Red111^T^, were isolated for further analysis. Unless mentioned otherwise, the isolates in this study were cultured on modified R2A agar or in degassed R2A broth (Wako, Japan) with 20 mM fumarate (modified R2A broth). The four strains were preserved at −80°C in modified R2A broth with 10% (v/v) dimethyl sulfoxide. *Geobacter bemidjiensis* DSM 16622^T^, obtained from the Leibniz-Institut Deutsche Sammlung von Mikroorganismen und Zellkulturen (DSMZ), was used as a reference strain in this study.

### Phylogenetic and Genomic Analyses

For phylogenetic analysis, the almost complete 16S rRNA genes of the four strains were amplified using primers 27F and 1492R and sequenced using primers 27F, 926F, 519R, and 1492R as described by Ashida et al. (2010). The similarities of 16S rRNA genes between the four strains and their phylogenetic neighbors were determined using the identify service from the EzBioCloud Database^2^ (Yoon et al., 2017). The 16S rRNA gene sequences of related strains were downloaded from the NCBI database and aligned using the CLUSTAL W algorithm in MEGA version 7.0 (Kumar et al., 2016). Genetic distances and clustering were determined using Kimura 2-parameter model (Kimura, 1980), and phylogenetic trees were reconstructed by the neighbor-joining method (NJ). Trees were also reconstructed using the maximum-likelihood (ML) and maximum-parsimony (MP) methods to ensure the robustness of the conclusion. ML trees were reconstructed using the best-fit substitution model Kimura 2-parameter + G + I, while MP trees were reconstructed with the default Subtree-Pruning-Regrafting (SPR) method. Bootstrap values were evaluated based on 1000 replicates in the three methods. In order to elucidate the exact taxonomic positions of the four strains, a phylogenetic tree based on 92 concatenated core genes was also constructed using the UBCG pipeline^3^ as described previously with default parameters (Na et al., 2018). The sequences of 92 core genes were retrieved from the draft genome sequences as described in the next paragraph. In addition, previous studies revealed that five housekeeping genes (*fusA*, *gyrB*, *nifD*, *recA*, and *rpoB*) are especially useful for defining the phylogeny of the family *Geobacteraceae* (Holmes et al., 2004), so a MLSA of these five housekeeping genes was performed with the primers GYR48F/GYR1010R (*gyrB*, 930 bp in length), NIFDF2/NIFDR2 (*nifD*,768 bp in length), RPO175F/RPO800R (*rpoB*, 597 bp in length) and two modified primer pairs for *recA* (recF: 5′-TGATCGAGATCTTCGGACCGGA-3′; recR: 5′-TCCTTGCCGTAGGAGAACCA-3′, 672 bp in length) and *fusA* (fusF: 5′-CTNGACATCAAGATCTGCCC-3′; fusR: 5′-TTCGCCTCNACCTTGAACTC-3′, 642 bp in length). The deduced amino acid sequences of the five housekeeping genes were translated with standard codons using MEGA 7.0 software (Kumar et al., 2016). After the nucleotide sequences and amino acid sequences of the five housekeeping genes were aligned and concatenated in the order *rpoB*–*recA*–*nifD*–*gyrB*–*fusA*, phylogenetic trees based on concatenated nucleic acid and amino acid sequences were then generated using the ML method with the best-fit substitution models.

Genomic DNA was extracted from 5-day-old cultures grown on modified R2A agar using the DNeasy Blood and Tissue Kit (Qiagen, Germany), according to the manufacturer’s instructions for Gram-negative bacteria. The draft genome sequences of the four analyzed strains (S43^T^, Red53^T^, S62^T^, and Red111^T^) were generated using the Illumina Hiseq 2500 platform with massively parallel sequencing (MPS) Illumina technology at the Beijing Novogene Bioinformatics Technology Co. Ltd. (Beijing, China). The cleaned data was then assembled using the SOAP *de novo* version 2.04 (Li et al., 2008) and GapCloser version 1.12. Genomic G + C content calculations and functional gene annotations were performed based on assembled contigs using RAST Server with ClassicRAST annotation scheme (Aziz et al., 2008). The whole genome sequences of the type or reference strains of the known *Geobacter* species were obtained from NCBI database. The genome similarities of the nucleotide sequences among the four novel strains and other type strains in the family *Geobacteraceae* were determined with the ANI and GGDC values. ANI values were obtained using JspeciesWS based on the recommended BLAST + alignment^4^ (Richter et al., 2016), while GGDC values from the Genome-to-Genome Distance Calculator 2.1 under recommended BLAST + alignment and Formula 2^5^ (Meier-Kolthoff et al., 2013). In order to assess the genus affiliations, the AAI and the POCP were also used for amino acid-level comparisons for every pairwise combination of genomes. The AAI values were obtained by calculating the mean protein sequence similarity of all protein-coding genes shared between strains with an online-tool of Environmental Microbial Genomics Laboratory^6^ as described by Konstantinidis and Tiedje (2005). The POCP values were calculated using a Python script^7^ with the formula [(C1 + C2)/(T1 + T2)] × 100%, where C1 and C2 represent the conserved number of proteins in the two genomes being compared, respectively, and T1 and T2 represent the total number of proteins in the two genomes being compared, respectively (Qin et al., 2014).

In addition, a visual genomic comparison across strains S43^T^, Red53^T^, S62^T^, Red111^T^, and reference strain *G. bemidjiensis* DSM 16622^T^ at the nucleotide level was generated using the BLAST Ring Image Generator (BRIG) with default parameters (Alikhan et al., 2011). The genome of strain S43^T^ was used as the reference genome. For experimental genomic difference, genomic DNA fingerprints were carried out using the RAPD method (primer: AGCAGCGTGG) (Yu and Pauls, 1992) and rep-PCR with ERIC primers (ERIC1: 5′-ATGTAAGCT CCTGGGGATTCAC-3′; ERIC2: 5′-AAGTAAGTGACTGG GGTGAGCG-3′) and BOX primer (BoxA1R: 5′-CTA CGGCAAGGCGACGCTGACG-3′) (Versalovic et al., 1994; Ishii and Sadowsky, 2009).

### Phenotypic Characterization

Cell morphology was examined by transmission electron microscopy (model JEM-1400, JEOL) at 80 kV, after the cultures were negatively stained with ammonium molybdate. Colony size, shape, and color were determined after 3 days of incubation at 30°C on modified R2A agar. The temperature and pH tests were performed in modified R2A broth at different temperatures (6, 10, 13, 16, 20, 25, 30, 33, 37, 40, and 42°C) and different pH (adjusted from 5.0 to 9.0 in increments of 0.5 pH units) with 20 mM buffers (MES, pH 5.0–6.5; HEPES, pH 7.0–8.0, Tricine, pH 8.5–9.0). Growth was measured at OD_600_ using a spectrophotometer (Jasco V550, Japan) after 1 week of culture. The requirement and tolerance to NaCl was tested on modified R2A agar supplemented with various concentrations of NaCl (adjusted from 0 to 1.0% with 0.1% increments). To calculate the bacterial mean generation time of the four isolated strains, the growth curves were created using 30 mL modified R2A medium in 50 mL serum bottles at 30°C with 1/100 inoculation scale, and then the values were obtained under bacterial exponential phase as described by Todar’s Online Textbook of Bacteriology^8^. Electron donor and acceptor utilization tests were carried out using degassed MFM at 30°C. Acetate (10 mM) was used as the electron donor for all electron acceptor tests while Fe(III)-NTA (5 mM) was the electron acceptor for all electron donor tests. The final concentrations of detected electron acceptors and donors were utilized as previously described (Nevin et al., 2005). Enzymatic activities of the four isolates and reference strain were detected using API ZYM strips (bioMérieux) based on the manufacturer’s instructions.

Cytochrome analysis of the four isolated strains was performed with cell grown in MFM with 20 mM fumarate and 20 mM acetate. Cultures (5 mL) were collected and resuspended in 2 mL of 20 mM PIPES buffer (pH 7.0). A dithionite-reduced minus air-oxidized difference spectrum of whole cells was obtained using a Jasco V550 spectrophotometer (Lovley et al., 1993). The ferric reduction abilities of the four isolated strains were measured using 20 mL MFW medium in 50 mL serum bottles supplemented with 10 mM acetate and 8 mM Fe(III)-NTA under an 80:20 mixture gas of N_2_:O_2_. The vessels were incubated at 30°C without shaking and every set of experiments were performed in triplicate. After cultured samples were collected at different sampling time, the soluble ferrous concentration was detected immediately using a ferrozine buffer as described by Onley et al. (2018), as well as total iron content after mixed with equal volume of 10% (w/v) hydroxylamine hydrochloride solution. The soluble ferric concentration was calculated by subtracting the amount of ferrous from the total iron amount. Absorbance was measured using a Jasco V550 spectrophotometer at a wavelength of 562 nm. Standards were prepared with ferrous ammonium sulfate hexahydrate spanning a concentration range of 0.1–10 mM ferrous ions.

### Chemotaxonomic Characterization

For fatty acid detection, biomass of the four isolates and *G. bemidjiensis* DSM 16622^T^ was collected from cultures grown for 3 days at 30°C in modified R2A broth, when the bacteria were in the late exponential growth phase. After washed twice in autoclaved distilled water and freeze dried, the fatty acids were extracted as described previously (Kuykendall et al., 1988). The fatty acid profiles were determined using a gas chromatography mass spectrometry (GCMS-QP2010 ultra, Shimadzu, Japan) with the standard reagents for different fatty acids (Gunina et al., 2014). Biomass collection for respiratory quinones analysis was same as fatty acid detection, except cultured for 5 days to get more biomass. Extraction and purification were performed as described previously (Bligh and Dyer, 1959) and Sep-Pak plus silica (Waters, United States) was employed for separation of quinones. Quinone profiles were determined using HPLC with ACQUITY UPLC H-Class system (Waters, United States), which was carried out by the TechnoSuruga Laboratory (Shizuoka, Japan). The whole-cell protein profiles of the four isolates and *G. bemidjiensis* DSM 16622^T^ were detected using a matrix-assisted laser desorption/ionization time-of-flight mass spectrometry (MALDI-TOF MS) (AB SCIEX 5800, United States) according to the standard protocol described by Dridi and Drancourt (2011).

### Accession Numbers

The GenBank accession number of the 16S rRNA gene sequences of strains S43^T^, Red53^T^, S62^T^, and Red111^T^ are MH915553, MH915554, MH915555, and MH915556, respectively. Accession number of the *fusA* gene sequences are MK018132, MK018133, MK018134, and MK018135, respectively. Accession number of the *nifD* gene sequences are MK018136, MK018137, MK018138, and MK018139, respectively. Accession number of the *recA* gene sequences are MK018140, MK018141, MK018142, and MK018143, respectively. Accession number of the *rpoB* gene sequences are MK018144, MK018145, MK018146, and MK018147, respectively. Accession number of the *gyrB* gene sequences are MK018148, MK018149, MK018150, and MK018151, respectively.

This Whole Genome Shotgun project of strains S43^T^, Red53^T^, S62^T^ and Red111^T^, have been deposited at DDBJ/ENA/GenBank under the accession RAHW00000000, SSYB00000000, SSYA00000000, and SRSC00000000, respectively.

## Results and Discussion

### Phylogenetic Analysis

**FIGURE 1:**
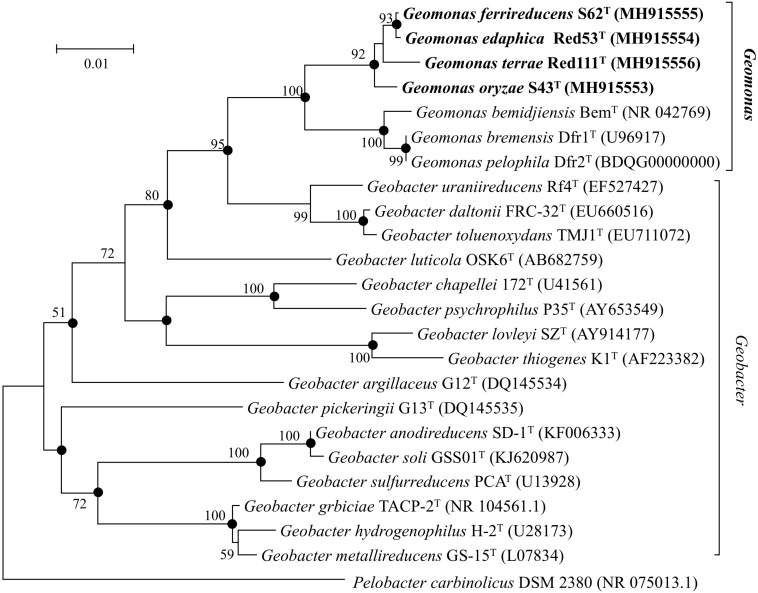
Neighbor-joining (NJ) phylogenetic tree based on 16S rRNA gene sequences showing the relationship of four novel strains S43^T^, Red53^T^, S62^T^, Red111^T^ with other type species in the family *Geobacteraceae*. The tree was reconstructed using MEGA 7.0 with Kimura 2-parameter model. Closed circles indicate branches that were recovered with all three tree-making methods (maximum-likelihood, maximum-parsimony, and neighbor-joining). Bootstrap values (expressed as percentages of 1,000 replications) over 50% are shown at branching nodes. Bar, 0.01 substitutions per nucleotide position.

**FIGURE 2:**
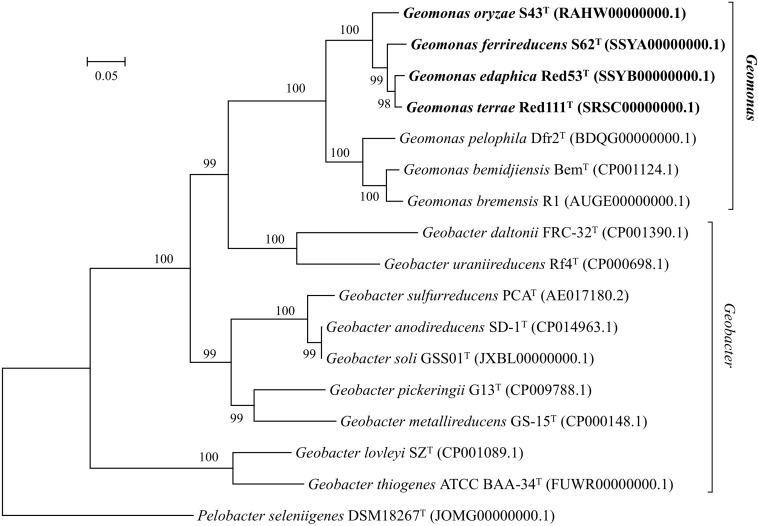
Maximum-likelihood (ML) phylogenetic tree based on the whole genome sequences showing the position of strains S43^T^, Red53^T^, S62^T^, Red111^T^ and representatives of some related taxa in the family *Geobacteraceae*. The tree was constructed using RAxML tool with GTR + CAT model based on concatenated alignment of 92 core genes (*alaS, argS, aspS, cgtA, coaE, cysS, dnaA, dnaG, dnaX, engA, ffh, fmt, frr, ftsY, gmk, hisS, ileS, infB, infC, ksgA, lepA, leuS, ligA, nusA, nusG, pgk, pheS, pheT, prfA, pyrG, recA, rbfA, rnc, rplA, rplB, rplC, rplD, rplE, rplF, rplI, rplJ, rplK, rplL, rplM, rplN, rplO, rplP, rplQ, rplR, rplS, rplT, rplU, rplV, rplW, rplX, rpmA, rpmC, rpmI, rpoA, rpoB, rpoC, rpsB, rpsC, rpsD, rpsE, rpsF, rpsG, rpsH, rpsI, rpsJ, rpsK, rpsL, rpsM, rpsO, rpsP, rpsQ, rpsR, rpsS, rpsT, secA, secG, secY, serS, smpB, tig, tilS, truB, tsaD, tsf, uvrB, ybeY, ychF*). Bootstrap values (expressed as percentages of 100 replications) over 70% are shown at branching nodes. Bar, 0.05 substitutions per nucleotide position.

**FIGURE 3:**
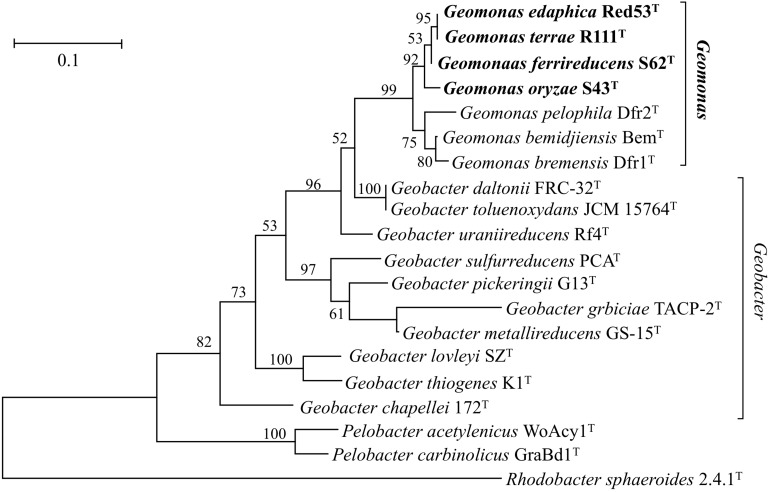
Maximum-likelihood (ML) phylogenetic tree showing the position of strains S43^T^, Red53^T^, S62^T^, Red111^T^ and representatives of some related taxa in the order *Desulfuromonadales* based on deduced amino acid sequences of five concatenated housekeeping gene: *rpoB* (1–200 amino acids), *recA* (201–425 amino acids), *nifD* (426–687 amino acids), *gyrB* (688–984 amino acids) and *fusA* (985–1180 amino acids). The tree was reconstructed using MEGA 7.0 with LG + G model. Bootstrap values (expressed as percentages of 1,000 replications) over 50% are shown at branching nodes. Bar, 0.1 substitutions per amino acid position.

Nearly complete 16S rRNA gene sequences were obtained for strain S43^T^ (1424 bp, accession number MH915553), strain Red53^T^ (1424 bp, accession number MH915554), strain S62^T^ (1424 bp, accession number MH915555), and strain Red111^T^ (1426 bp, accession number MH915556). Pairwise comparisons of the 16S rRNA gene sequences revealed 99.0–99.9% similarity between each pair within the four isolated strains. *G. bemidjiensis* Bem^T^ and *G. bremensis* Dfr1^T^ were the most closely related strains in the family *Geobacteraceae* to the four isolated strains and showed 16S rRNA gene sequence similarities of 97.4–98.2% and 97.1–98.0%, respectively. In the phylogenetic tree based on the 16S rRNA gene sequences, the four strains fell within the clade comprising species of the family *Geobacteraceae* and formed a coherent cluster with *G. bemidjiensis* Bem^T^, *G. bremensis* Dfr1^T^, and *G. pelophilus* Dfr2^T^, with a bootstrap resampling value of 100% (Figure 1). This result was confirmed by the phylogenetic trees drawn using the UBCG and MLSA, which placed the four strains S43^T^, S62^T^, Red111^T^, and Red53^T^ in an independent and monophyletic branch together with three relatives in the family *Geobacteraceae* (Figures 2, 3 and Supplementary Figure 1). These robust phylogenetic relationships suggest that the four newly isolated strains along with the other three phylogenetic relatives may consist of a novel taxon in the family *Geobacteraceae*.

Yarza et al. (2014) recently proposed that the generally used arbitrary genus threshold of 16S rRNA gene identity should be revised to a lower minimum value of 94.5% from the previous 95% with a confidence interval of 94.55–95.05 and median sequence identity of 96.4%. However, comparison of the 16S rRNA genes in the four novel type strains with the sequence of the type strain of the type species *G. metallireducens* showed an identity value of 92.4%, which is clearly lower than the common threshold for genus differentiation. Thus, together with the distinct branches on the phylogenetic trees, the four strains may be classified into a distinct genus from the type species *G. metallireducens*.

### Genome Analysis

The draft genome sizes of the four analyzed strains S43^T^, Red53^T^, S62^T^, and Red111^T^ were 4.93 Mb, 4.76 Mb, 4.81 Mb, and 4.70 Mb, which contained 4170, 4072, 4098, and 3959 annotated protein-coding open reading frames (ORFs), respectively. The genomic G + C content of the four analyzed strains were 60.6–61.2 mol%, which was in line with that of three phylogenetic relatives, *G. bemidjiensis* Bem^T^, *G. bremensis* Dfr1^T^, and *G. pelophilus* Dfr2^T^, showing values of 60.3–60.9 mol%, but obviously higher than those of other *Geobacter* species (Table 1; Nevin et al., 2005; Viulu et al., 2013). The detailed genomic characteristics of the four analyzed strains are listed in Supplementary Table 1. The ANI and GGDC values based on the genomic sequences of the four isolates and other *Geobacteraceae* species showed relatedness values below 77.6% and 22.8%, respectively (Table 2), which are well-below the defined thresholds for species delineation of 95–96% for ANI and 70% for GGDC (Chun et al., 2018), and support the inclusion of the four strains as a novel taxa in the family *Geobacteraceae*. When the four novel isolates were compared, the ANI values and GGDC values were in the range of 91.2–95.3% and 44.9–64.6%, respectively (Table 2). Among them, the maximum relatedness values related to strains S43^T^ and S62^T^ were 93.8% (ANI) and 56.2% (GGDC), which are lower than the threshold for delineation of bacterial species as described above, supporting the classification of strains S43^T^ and S62^T^ as two novel species. However, there was slightly more variability in strains Red53^T^ and Red111^T^ in the ANI and GGDC values (95.3% and 64.6%), respectively; the values are located in the transition zone for novel species description of 95–96% for ANI and 60–70% for GGDC (Richter and Rosselló-Móra, 2009). Although strains Red53^T^ and Red111^T^ can be separated from other known *Geobacteraceae* species as novel species with lower ANI (Max. 93.8%) and GGDC (Max. 56.2%) values, their species status cannot be separated from each other based on their genomic relatedness. Given the obscure position of strains Red53^T^ and Red111^T^, more comparisons were performed based on their physiological and biochemical characteristics.

**TABLE 1:** Differential characteristics between the four novel species and the type strains of phylogenetically related species of the family *Geobacteraceae*.

**Characteristic**	**1**	**2**	**3**	**4**	**5**	**6**	**7**	**8**	**9**	**10**	**11**
Optimal temperature (°C)	30–33	30–33	30–33	30–33	30	30–32	30–32	32	30	25–32	30–35
Optimal pH	6.0–7.0	6.0–7.0	6.0–7.0	6.0–7.0	7.0	5.5–6.7	6.7–7.0	6.5–7.0	6.7–7.3	6.6–7.0	ca. 6.7
Motility	+	+	+	+	−	−	−	+	ND	−	−
Electron acceptor usage											
Malate	+	−	−	+	+	+	+	+	+	ND	ND
Nitrate	+	+	+	+	−	ND	ND	−	−	−	+
Sulfur	−	−	−	−	−	+	+	−	+	−	−
MnO_2_	−	−	−	−	+	+	+	+	ND	−	+
Fe(III) citrate	−	−	−	−	+	+	+	−	+	+	+
Electron donor usage											
Fumarate	+	+	+	+	+	+	+	+	+	−	−
Succinate	+	+	+	+	+	+	+	−	−	−	−
Butanol	−	−	−	−	−	+	−	−	+	ND	+
Propionate	+	+	+	+	+	+	+	−	−	+	+
Malate	+	+	+	+	+	+	+	+	ND	−	−
Lactate	+	+	+	+	+	+	−	+	−	−	−
Methanol	+	−	+	+	−	ND	ND	−	ND	ND	−
Benzaldehyde	−	−	−	−	−	ND	ND	ND	ND	+	+
Toluene	−	−	−	−	−	ND	ND	ND	+	+	+
Phenol	−	−	−	−	−	ND	ND	ND	ND	+	+
G + C content (mol%)	61.2	60.5	60.7	61.0	60.3	60.0	60.9^∗^	54.0	53.0	54.4	56.6

*(1) *Geomonas oryzae* S43^*T*^ (data from this study); (2) *Geomonas edaphica* Red53^*T*^ (data from this study); (3) *Geomonas ferrireducens* S62^*T*^ (data from this study); (4) *Geomonas terrae* Red111^*T*^ (data from this study); (5) *Geobacter bemidjiensis* DSM 16622^*T*^ (data from this study); (6) *Geobacter bremensis* Dfr1^*T*^ (data from Straub and Buchholz-Cleven, 2001); (7) *Geobacter pelophilus* Dfr2^*T*^ (data from Straub and Buchholz-Cleven, 2001); (8) *Geobacter uraniireducens* Rf4^*T*^ (data from Shelobolina et al., 2008); (9) *Geobacter daltonii* FRC-32^*T*^ (data from Prakash et al., 2010); (10) *Geobacter toluenoxydans* TMJ1^*T*^ (data from Kunapuli et al., 2010); (11) *Geobacter metallireducens* GS-15^*T*^ (data from Lovley et al., 1993 and Zhou et al., 2014). +, Positive; −, negative. ND, no data. ^∗^Data taken from genome analysis based on NCBI database.*

**TABLE 2:** Average nucleotide identity (ANI) and Genome-to-Genome-Distance (GGDC) comparisons between the four novel strains S43^T^, Red53^T^, S62^T^, Red111^T^ and other type species in the family *Geobacteraceae*.

**Reference strains**	**Accession numbers of genomic data^#^**	**ANI value (%)**					**GGDC value (%)**		
			
		**S43^T^**	**Red53^T^**	**S62^T^**	**Red111^T^**	**S43^T^**	**Red53^T^**	**S62^T^**	**Red111^T^**
*Geomonas oryzae* S43^T^	RAHW00000000	100				100			
*Geomonas* edaphica Red53^T^	SSYB00000000	91.2	100			44.9	100		
*Geomonas ferrireducens* S62^T^	SSYA00000000	91.8	93.2	100		47.5	54.4	100	
*Geomonas terrae* Red111^T^	SRSC00000000	91.9	95.3	93.8	100	47.2	64.6	56.2	100
*Geobacter pelophilus* Dfr2^T^	BDQG01000001.1	77.5	77.8	77.3	77.3	22.7	22.6	22.6	22.4
*Geobacter bemidjiensis* Bem^T^	NC_011146.1	77.5	77.1	77.2	77.1	22.6	22.3	22.3	22.2
*Geobacter bremensis* R1	AUGE01000001.1	77.6	77.2	77.3	77.2	22.8	22.5	22.4	22.3
*Geobacter metallireducens* GS-15^T^	NC_007517.1	70.7	70.6	70.8	70.5	19.4	19.3	19.1	18.9
*Geobacter uraniireducens* Rf4^T^	NC_009483.1	70.4	70.4	70.4	70.4	20.1	19.1	19.2	18.8
*Geobacter lovleyi* SZ^T^	NC_010814.1	68.0	68.0	67.9	68.0	18.7	18.9	19.3	17.0
*Geobacter toluenoxydans* JCM 15764^T^	BBCJ01000001.1	68.4	68.2	68.6	68.2	23.2	21.4	21.4	22.8
*Geobacter daltonii* FRC-32^T^	NC_011979.1	69.1	69.1	69.3	69.0	19.4	19.3	18.8	18.4
*Geobacter sulfurreducens* PCA^T^	NC_002939.5	70.0	70.1	70.1	70.0	18.6	18.3	18.6	18.4
*Geobacter thiogenes* ATCC BAA-34^T^	FUWR01000042.1	67.5	67.4	67.5	67.4	19.8	18.9	19.7	17.5
*Geobacter anodireducens* SD-1^T^	NZ_CP014963.1	70.2	70.3	70.4	70.3	18.7	18.5	18.7	18.5
*Geobacter pickeringii* G13^T^	NZ_CP009788.1	71.2	71.1	71.2	71.1	19.3	19.2	19.6	19.2
*Geobacter soli* GSS01^T^	NZ_CP009788.1	70.3	70.3	70.4	70.2	18.7	18.4	18.7	18.4

*^#^Draft genomes or chromosome sequences of all reference strains were retrieved from NCBI database.*

**FIGURE 4:**
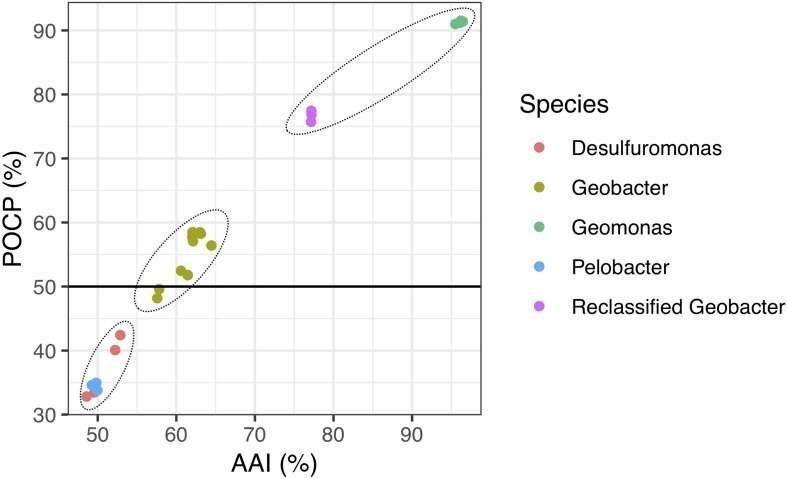
Relationship between AAI and POCP values from pairwise whole-genome comparisons. Each dot represents an average comparison value between the four novel strains and their closely relatives in the order *Desulfuromonadales.* The circles indicate the three different groups separated by AAI and POCP values. A total of 24 genomes were included in this analysis. “Reclassified *Geobacter*” contains *G. bemidjiensis*, *G. bremensis*, and *G. pelophilus*.

Because genomic relatedness with nucleotide sequences were not suitable for genera separation (Qin et al., 2014), we further determined AAI and POCP values, which are robust approaches based on protein sequences, to determine the genome distance between the four strains and other known closely strains in the order *Desulfuromonadales* (Figure 4 and Supplementary Table 2). The four strains shared similarities of 77.0–77.4% for AAI and 75.6–77.8% for POCP to *G. bemidjiensis*, *G. pelophilus*, and *G. bremensis*, 57.5–64.5% for AAI and 48.0–59.1% for POCP to other known *Geobacter* species, and 48.6–53.0% for AAI and 33.2–42.5% for POCP to the genera *Pelobacter* and *Desulfuromonas.* The two genera *Pelobacter* and *Desulfuromonas*, used for comparison, were the closest phylogenetic neighbors to the four isolates except for the genus *Geobacter*, although they belong a different family *Desulfuromonadaceae* (Greene et al., 2009). Qin et al. (2014) proposed a threshold for genera delineation with the 50% of POCP, meaning the four strains belong to different genera from *G. lovleyi* and *G. thiogenes* of the family *Geobacteraceae*. However, based on 16S rRNA gene phylogenetic analysis, the type strains of *G. lovleyi* and *G. thiogenes* did not show the furthest genetic distance from the four strains within the family *Geobacteraceae*. The 50% boundary of POCP, hence, is not an appropriate metric for delineating genera within the family *Geobacteraceae*, as reported previously for the families *Methylococcaceae* (Orata et al., 2018), *Bacillaceae* (Aliyu et al., 2016), *Burkholderiaceae* (Lopes-Santos et al., 2017), *Neisseriaceae* (Li et al., 2017), and *Rhodobacteraceae* (Wirth and Whitman, 2018). Additionally, AAI, which delimits taxonomic ranks at the genus levels, has a proposed a threshold range of 60–80% to separate different genera (Luo et al., 2014; Rodriguez-R and Konstantinidis, 2014). However, because of the large range in genera separation, specific AAI boundaries have been proposed for several families for genera delineation. For example, the family *Methylococcaceae* used 71% AAI as the lower genus limit (Orata et al., 2018), the family *Methylothermaceae* used 70% AAI as the threshold to separate its genera (Skennerton et al., 2015), and ∼80% AAI was reported for the family *Rhodobacteraceae* (Wirth and Whitman, 2018). Thus, the large difference from 64.6% AAI (maximum value within *Geobacter* species) to 77.0% AAI (minimum value within proposed *Geomonas* species) is distinct enough to define two different genera in the family *Geobacteraceae*, particularly under the condition in which the minimum AAI difference between the two different families was only 5% (Supplementary Table 2).

**FIGURE 5:**
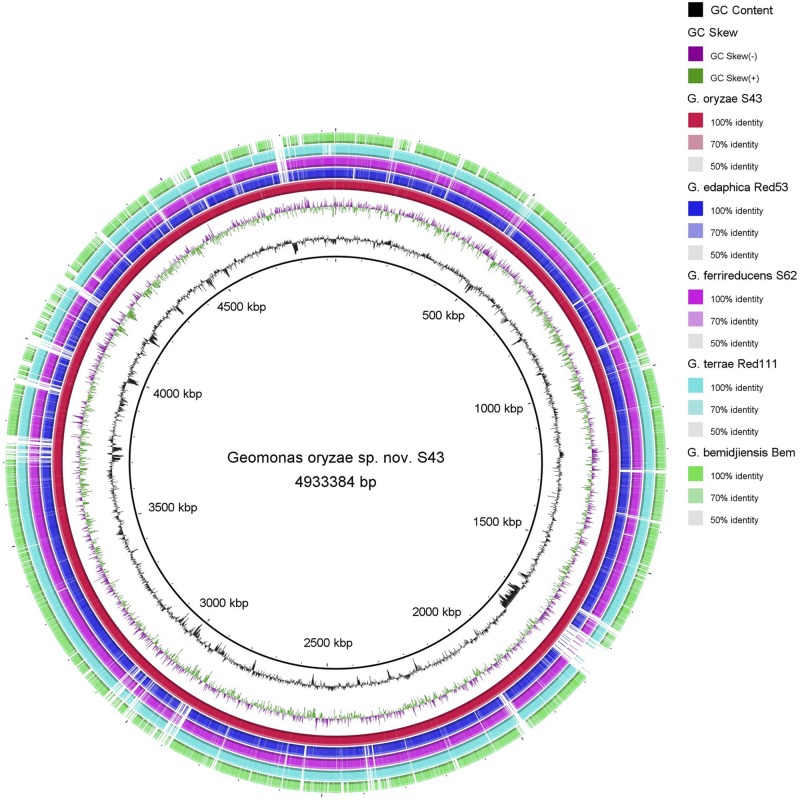
Circular representation of whole-genome sequences of *Geomonas oryzae* S43^T^, *Geomonas edaphica* Red53^T^, *Geomonas ferrireducens* S62^T^, *Geomonas terrae* Red111^T^ and *Geobacter bemidjiensis* DSM 16622^T^. The rings from inner to outer: ring 1–G + C content, ring 2–GC skew, ring 3–whole-genome sequences of *G. oryzae* S43^T^ (red), ring 4–whole-genome sequences of *G. edaphica* Red53^T^ (blue), ring 5–whole-genome sequences of *G. ferrireducens* S62^T^ (pink), ring 6–whole genome sequences of *G. terrae* Red111^T^ (cyan), ring 7–whole-genome sequences of *G. bemidjiensis* DSM 16622^T^ (green). Comparison created using BRIG platform application.

**FIGURE 6:**
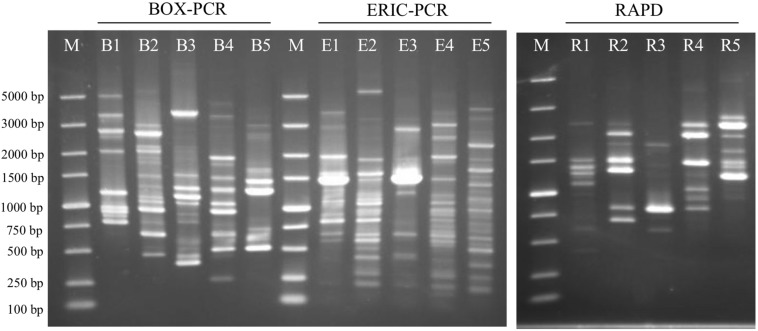
Agarose gel-electrophoresis with rep-PCR and RAPD patterns from the analyzed five strains. BOX-PCR indicates rep-PCR with BOX primer and ERIC-PCR indicates rep-PCR with ERIC primers. B1, E1 and R1: *Geomonas oryzae* S43^T^; B2, E2 and R2: *Geomonas edaphica* Red53^T^; B3, E3 and R3: *Geomonas ferrireducens* S62^T^; B4, E4 and R4: *Geomonas terrae* Red111^T^; B5, E5 and R5: *Geobacter bemidjiensis* DSM 16622^T^; M: DNA marker.

The BRIG analysis indicated that most regions within the five analyzed genomes were conserved with at least 70% similarity (Figure 5). Furthermore, the four novel isolates (S43^T^, Red53^T^, S62^T^, and Red111^T^) showed higher similarity with each other in most genomic regions compared with the reference strain genome, indicating that the four novel strains are more closely related to each other than to *G. bemidjiensis* DSM 16622^T^, which is consistent with the results of the above phylogenetic and genomic analyses (Figure 2 and Table 2). Genomic DNA fingerprints of the four isolated strains and the reference strain *G. bemidjiensis* DSM 16622^T^ showed clearly different banding patterns (Figure 6), confirming that the four isolates are not of clonal origin and distinct from each other.

### Morphological and Physiological Characterization

**FIGURE 7:**
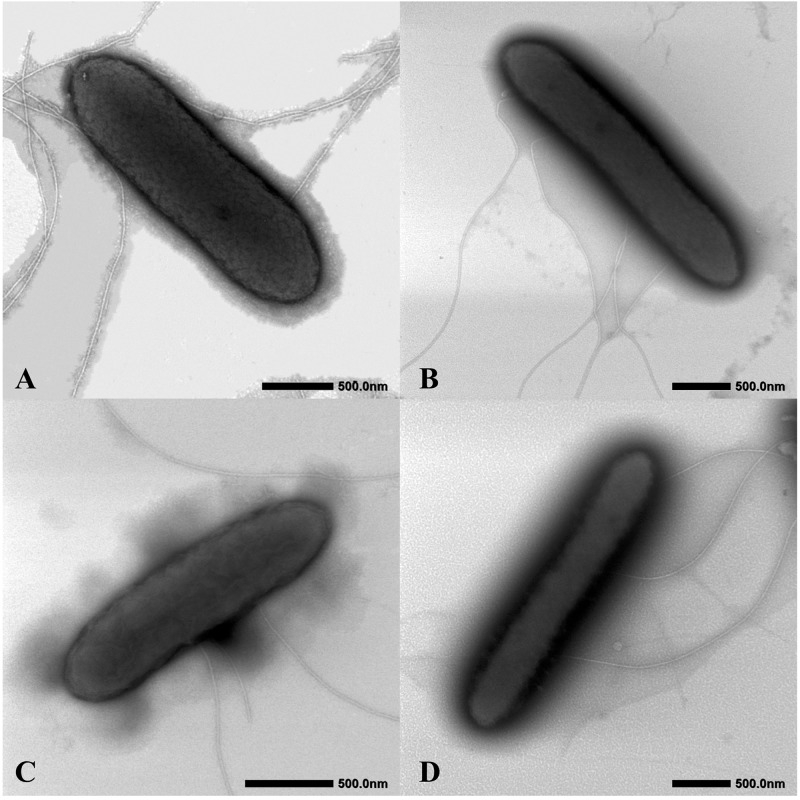
Cellular transmission electron micrograph of the four isolated strains. Cells were grown on R2A agar supplemented with 20 mM fumarate at 30°C for 5 days. **(A)** *Geomonas oryzae* S43^T^; **(B)** *Geomonas edaphica* Red53^T^; **(C)** *Geomonas ferrireducens* S62^T^; **(D)** *Geomonas terrae* Red111^T^. Bar, 500 nm.

**FIGURE 8:**
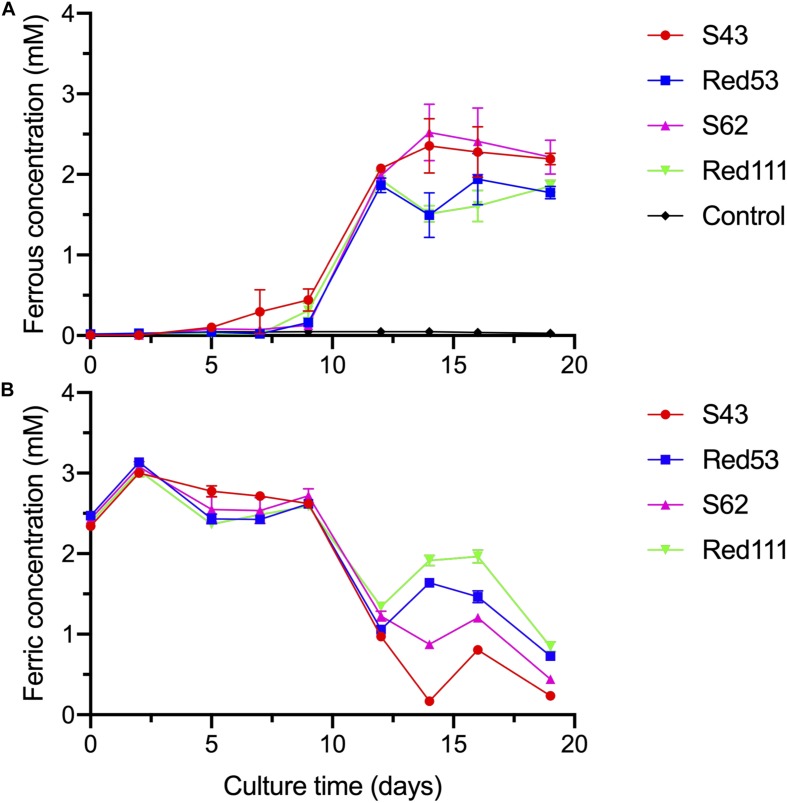
Reduction of Fe(III)-NTA by the four isolated strains at different culture time, with acetate as the electron donor. **(A)** dissolved ferrous concentration; **(B)** dissolved ferric concentration. Control means no-bacteria medium. Data were all presented as means ± standard deviations (SD) of triplicate. When not shown, error bars are smaller than the symbol size.

The four isolates were characterized as Gram-stain negative, motile, and strictly anaerobic. Growth on the modified R2A plate were better than on the modified freshwater plate. TEM images of the four isolates showed that the cells were rod-shaped, with 0.4–0.8 μm wide and 1.0–2.5 μm long and contained peritrichous flagella for motility (Figure 7). Growth of the four strains was observed under 42°C with an optimum of 30–33°C and at pH 5.5–8.0 with an optimum of pH 6.0–7.0, whereas strains Red53^T^ and Red111^T^ did not grow below 10°C; strains S43^T^ and S62^T^ did not grow below 13°C. All the four strains can tolerate 0.7% (w/v) NaCl and grew well under 0.2%. Based on the bacterial growth curves (Supplementary Figure 2), the mean generation times of the four isolated strains S43^T^, Red53^T^, S62^T^, and Red111^T^ were 206, 271, 237, and 271 min, respectively. For enzyme activity detected with API ZYM strips, all analyzed strains contained leucine arylamidase, acid phosphatase, and naphthol-AS-BI-phosphohydrolase activities. Besides them, strains S43^T^ and S62^T^ contained esterase (C4) and esteraselipase (C8) activities, strain Red53^T^ also contained alkaline phosphatase, esterase (C4), and esteraselipase (C8) activities, and *G. bemidjiensis* DSM 16622^T^ contained esterase (C4) activity. The *c*-type cytochromes were present based on the different spectra of whole cells (Supplementary Figure 3). The absorbance peaks for strains S43^T^, S62^T^, and Red111^T^ were 424, 524, and 554 nm, with slight differences observed for Red53^T^ (424, 522, and 554 nm), but distinct differences from the reference strain *G. bemidjiensis* DSM 16622^T^ (422, 522, and 555 nm) (Nevin et al., 2005). Similar to other species in the family *Geobacteraceae*, the four isolated strains possessed ferric reducing ability, which reduced most ferric iron [Fe(III)-NTA] to ferrous iron in 20 days, although the reduction rates for the four strains were not identical (Figure 8). Other phenotypic characteristics of the strains are presented in Table 1 along with the species description. Based on these physiological features, strain Red53^T^ was clearly differed from the other three isolates, confirming the possible distinct species status from strain Red111^T^, the closest genomic relative.

### Chemotaxonomic Characterization

The major fatty acids (>10% of the total fatty acids) present in strain S43^T^ were identified as iso-C_15__:__0_ and C_15__:__0_ 3-OH, which was the same as strains S62^T^ and Red111^T^, but different from strain Red53^T^ and the reference strain *G. bemidjiensis* DSM 16622^T^, because iso-C_15__:__0_ and C_15__:__0_ were the major fatty acids for strain Red53^T^ and iso-C_15__:__0_, C_15__:__0_, and C_15__:__0_ 3-OH were for *G. bemidjiensis* DSM 16622^T^. Other moderate and minor amounts of the five strains showed no significant differences, as shown in Table 2. Notably, strain Red53^T^ showed a different major fatty acid profiles from the other three isolated strains, which further certified the separate species status of strain Red53^T^. The respiratory quinone was also investigated for the four isolates and MK-8 was identified as the predominant quinone, in line with the quinone profiles of other reported *Geobacteraceae* species (Viulu et al., 2013; Sun et al., 2014; Zhou et al., 2014). Small amounts of MK-7 and MK-9 were also detected for the four strains, among which the presence of MK-7 was in the first description of the family *Geobacteraceae* (Sun et al., 2014). In whole-cell protein profile analysis of the five strains by MALDI-TOF MS, the major peaks were located at m/z 5000–6000, whereas the exact position and content of each peak in different strains were obviously distinct (Supplementary Figure 4, asterisks), revealing differences in the whole-cell protein profiles and confirming the distinctiveness among the five strains.

## Conclusion

In this study, we isolated four novel strains (S43^T^, Red53^T^, S62^T^, and Red111^T^) from paddy soils collected from different fields in Japan. Phylogenetic analyses based on 16S rRNA genes, UBCG, and MLSA showed that the four strains formed a robust group together with three relatives (*G. bemidjiensis*, *G. bremensis*, and *G. pelophilus*) and were obviously separate from other type strains in the family *Geobacteraceae* (Figures 1–3 and Supplementary Figure 1). When strains S43^T^, S62^T^ and Red111^T^ were compared with each other or with their three closest phylogenetic relatives, the genomic relatedness with ANI and GGDC values were less than the proposed threshold value for species delineation (Table 2), indicating that the three isolated strains are not from a clonal origin and represent three novel species. This distinctiveness was also supported by the DNA fingerprints, MALDI-TOF maps, and fatty acid profile results (Figure 6, Table 3, and Supplementary Figure 4). When comparing strains Red53^T^ to Red111^T^, although they showed high genomic relatedness (Table 2) in the transition zone for novel species classification, their physiological and biochemical characteristics clearly differed. Thus, the four isolates represented four novel species in the family *Geobacteraceae*.

**TABLE 3:** Fatty acid compositions of the four novel strains and the reference strain *Geobacter bemidjiensis* DSM 16622^T^.

	**1**	**2**	**3**	**4**	**5**
iso-C_13__:__0_	2.9	ND	8.5	2.8	ND
C_13__:__0_	0.2	0.5	0.5	0.1	ND
iso-C_14__:__0_	1.6	1.5	1.2	1.2	0.6
C_14__:__0_	5.3	5.6	6.4	5.1	1.7
C_14__:__0_ 3-OH	1.5	1.0	2.1	2.3	1.0
iso-C_15__:__0_	**19.6**	**43.5**	**22.3**	**37.7**	**29.7**
a-C_15__:__0_	0.7	0.5	–	0.4	0.1
Unidentified (12.1)	1.6	–	0.3	1.5	0.4
C_15__:__0_	2.6	**12.8**	9.1	1.3	2.3
C_15__:__1_ ω5c	1.9	6.5	0.1	1.3	1.3
C_15__:__0_ 3-OH	**20.6**	9.1	**10.8**	**19.8**	**11.5**
Unidentified (13)	ND	ND	3.0	ND	ND
iso-C_16__:__0_	1.5	1.5	0.9	0.7	2.0
C_16__:__1_ ω9c	2.2	2.6	1.3	2.1	1.6
C_16__:__0_	7.6	8.2	9.4	4.5	**15.0**
C_16__:__1_ ω7c	7.1	0.8	5.5	6.3	8.7
C_16__:__0_ 10-methyl	5.0	0.3	ND	ND	6.4
C_16__:__0_ 3-OH	1.3	ND	5.1	ND	ND
C_16__:__1_ ω5c	1.6	0.8	0.8	1.2	1.4
iso-C_17__:__0_	1.1	0.6	0.9	0.4	3.9
Unidentified (15.4)	1.4	ND	0.3	0.9	0.7
a-C_17__:__0_	1.0	–	0.3	0.6	0.3
C_17__:__0_	0.2	0.5	0.6	–	0.6
C_17__:__0_ 3-OH	6.6	0.4	7.3	6.8	4.9
C_17__:__1_ ω7c	1.1	2.8	2.0	0.6	1.3
C_18__:__0_ 3-OH	2.5	–	0.8	1.3	2.8
C_18__:__1_ ω7c	–	–	–	0.2	0.6

*(1) *Geomonas oryzae* S43^*T*^; (2) *Geomonas edaphica* Red53^*T*^; (3) *Geomonas ferrireducens* S62^*T*^; (4) *Geomonas terrae* Red111^*T*^; (5) *Geobacter bemidjiensis* DSM 16622^*T*^. All data in this table come from this study, only those accounting for 0.5% or more in one of the strains are given. The dominant fatty acids are in bold. –, trace quantities (<0.1%). ND, not detected. Unidentified fatty acid with a retention time of 12.1, 13, and 15.4 min, respectively.*

In addition, the 16S rRNA gene similarities between the four strains and *G. metallireducens*, the type species of the genus *Geobacter*, showed a lower value than the common threshold for genus differentiation (Yarza et al., 2014). On the basis of the genomic G + C content, the four strains together with three relatives were separated clearly from their close phylogenetic neighbors, *G. uraniireducens*, *G. daltonii* and *G. toluenoxydans* with >5 mol% difference (Table 1). These facts, along with the robust phylogenetic positions and the difference in AAI values, indicate that the four isolated strains represent a novel genus in the family *Geobacteraceae*. Therefore, we proposed a new genus, *Geomonas oryzae* gen. nov., sp. nov., which also includes the new combinations *Geomonas bemidjiensis* comb. nov., *Geomonas bremensis* comb. nov., and *Geomonas pelophila* comb. nov. to accommodate *G. bemidjiensis*, *G. bremensis*, and *G. pelophilus*. Additionally, the phylogenetic trees based on gene and protein sequences strongly suggests that the remaining *Geobacter* is still paraphyletic (Figures 1–3). This proposition of the novel genus *Geomonas* is a pioneer but only a start of the taxonomic reclassification for the genus *Geobacter*. More taxonomic studies are needed to overhaul *Geobacter* taxonomy.

### Description of *Geomonas* gen. nov.

*Geomonas* (Ge.o.mo’nas. Gr. fem. n. *ge*, the earth; L. fem. n. *monas*, a unit, monad; N.L. fem. n. *Geomonas*, a monad from the earth).

Cells are Gram-stain negative, strictly anaerobic, chemoheterotrophic, non-spore-forming, rod-shaped, and motile by peritrichous flagella or non-motile. Colonies are red-pigmented due to the presence of c-type cytochromes. Able to use fumarate, Fe(III)-NTA, and ferrihydrite as the electron acceptors in the presence of a variety of electron donors, including acetate, succinate, ethanol, pyruvate, propionate, malate, and lactate. The major fatty acid is iso-C _15__:__0_. The predominant quinone is MK-8; MK-7 and MK-9 are also present in some species. The genomic DNA G + C content is 60.0-61.2 mol%.

The type species is *Geomonas oryzae.*

### Description of *Geomonas oryzae* sp. nov.

*Geomonas oryzae* (o.ry’zae. L. gen. n. *oryzae*, of rice, referring to the isolation of the type strain from rice paddy soil).

Besides the characteristics that define the genus, the following characteristics are observed. Growth occurs at 13–42°C (optimum, 30–33°C), at pH 5.5–8.0 (optimum, 6.0–7.0), and with 0–0.7% (w/v) NaCl (optimum, 0–0.2%). With Fe(III)-NTA as electron acceptor, yeast powder, mannitol, glucose, tryptone, casamino acid, methanol, glutamine, nicotinate, glycerol, isopropanol, serine, naphthalene, salicylic acid, proline, and arginine can also be utilized as electron donor, but phenol, butanol, toluene, benzyl alcohol, and benzaldehyde cannot be utilized as electron donor. With acetate as electron donor, malate, nitrite, and nitrate can also be utilized as electron acceptor, but Fe(III) pyrophosphate, Fe(III) citrate, sulfur, and MnO_2_ cannot be utilized as electron acceptor. Esterase (C4), esteraselipase (C8), leucine arylamidase, acid phosphatase, and naphthol-AS-BI-phosphohydrolase activities were present but alkaline phosphatase, lipase (C14), valine arylamidase, cystine arylamidase, trypsin, α-chymotrypsin, α-galactosidase, β-galactosidase, β-glucuronidase, α-glucosidase, β-glucosidase, *N*-acetyl-β-glucosaminidase, α-mannosidase, and α-fucosidase activities are absent. The major fatty acids are iso-C _15__:__0_ and C _15__:__0_ 3-OH. MK-7 and MK-9 are present in minor amounts.

The type strain, S43^T^ (= MCCC 1K03691^T^ = JCM 33030^T^), was isolated from paddy soil of a field in Niigata, Japan in February 2018. The genomic DNA G + C content of type strain is 61.2 mol%.

### Description of *Geomonas edaphica* sp. nov.

*Geomonas edaphica* (e.da’phi.ca. Gr. neut. n. *edaphos*, ground; L. fem. suff. -*ica*, adjectival suffix used with the sense of belonging to; N.L. fem. adj. *edaphica*, living in soil).

Besides the characteristics that define the genus, the following characteristics are observed. Growth occurs at 10–42°C (optimum, 30–33°C), at pH 5.5–8.0 (optimum, 6.0–7.0), and with 0–0.7% (w/v) NaCl (optimum, 0–0.2%). With Fe(III)-NTA as electron acceptor, yeast powder, mannitol, glucose, tryptone, casamino acid, glutamine, nicotinate, glycerol, isopropanol, serine, naphthalene, salicylic acid, proline, and arginine can also be utilized as electron donor, but methanol, phenol, butanol, toluene, benzyl alcohol, and benzaldehyde cannot be utilized as electron donor. With acetate as electron donor, nitrite and nitrate can also be utilized as electron acceptor, but malate, Fe(III) pyrophosphate, Fe(III) citrate, sulfur, and MnO_2_ cannot be utilized as electron acceptor. Esterase (C4), esteraselipase (C8), leucine arylamidase, alkaline phosphatase, acid phosphatase, and naphthol-AS-BI-phosphohydrolase activities were present but lipase (C14), valine arylamidase, cystine arylamidase, trypsin, α-chymotrypsin, α-galactosidase, β-galactosidase, β-glucuronidase, α-glucosidase, β-glucosidase, *N*-acetyl-β-glucosaminidase, α-mannosidase, and α-fucosidase activities are absent. The major fatty acids are iso-C _15__:__0_ and C _15__:__0_. MK-7 and MK-9 are present in minor amounts.

The type strain, Red53^T^ (= MCCC 1K04027^T^ = NBRC 114064^T^), was isolated from paddy soil of a field in Yamaguchi, Japan in March 2018. The genomic DNA G + C content of type strain is 60.5 mol%.

### Description of *Geomonas ferrireducens* sp. nov.

*Geomonas ferrireducens* (fer.ri.re.du’cens. L. neut. n. *ferrum*, iron; L. pres. part. *reducens*, converting to a different state; N.L. part. adj. *ferrireducens*, reducing ferric ions).

Besides the characteristics that define the genus, the following characteristics are observed. Growth occurs at 13–42°C (optimum, 30–33°C), at pH 5.5–8.0 (optimum, 6.0–7.0), and with 0–0.7% (w/v) NaCl (optimum, 0–0.2%). With Fe(III)-NTA as electron acceptor, yeast powder, mannitol, glucose, tryptone, casamino acid, methanol, glutamine, nicotinate, glycerol, isopropanol, serine, naphthalene, salicylic acid, proline, and arginine can also be utilized as electron donor, but phenol, butanol, toluene, benzyl alcohol, and benzaldehyde cannot be utilized as electron donor. With acetate as electron donor, nitrite and nitrate can also be utilized as electron acceptor, but malate, Fe(III) pyrophosphate, Fe(III) citrate, sulfur, and MnO_2_ cannot be utilized as electron acceptor. Esterase (C4), esteraselipase (C8), leucine arylamidase, acid phosphatase, and naphthol-AS-BI-phosphohydrolase activities were present but alkaline phosphatase, lipase (C14), valine arylamidase, cystine arylamidase, trypsin, α-chymotrypsin, α-galactosidase, β-galactosidase, β-glucuronidase, α-glucosidase, β-glucosidase, *N*-acetyl-β-glucosaminidase, α-mannosidase, and α-fucosidase activities are absent. The major fatty acids are iso-C _15__:__0_ and C _15__:__0_ 3-OH. MK-7 and MK-9 are present in minor amounts.

The type strain, S62^T^ (= MCCC 1K04028^T^ = NBRC 114031^T^), was isolated from paddy soil of a field in Niigata, Japan in February 2018. The genomic DNA G + C content of type strain is 60.7 mol%.

### Description of *Geomonas terrae* sp. nov.

*Geomonas terrae* (ter’rae. L. gen. n. *terrae* of/from soil).

Besides the characteristics that define the genus, the following characteristics are observed. Growth occurs at 10–42°C (optimum, 30–33°C), at pH 5.5–8.0 (optimum, 6.0–7.0), and with 0–0.7% (w/v) NaCl (optimum, 0–0.2%). With Fe(III)-NTA as electron acceptor, yeast powder, mannitol, glucose, tryptone, casamino acid, methanol, glutamine, nicotinate, glycerol, isopropanol, serine, naphthalene, salicylic acid, proline, and arginine can also be utilized as electron donor, but phenol, butanol, toluene, benzyl alcohol, and benzaldehyde cannot be utilized as electron donor. With acetate as electron donor, malate, nitrite, and nitrate can also be utilized as electron acceptor, but Fe(III) pyrophosphate, Fe(III) citrate, sulfur, and MnO_2_ cannot be utilized as electron acceptor. Leucine arylamidase, acid phosphatase, and naphthol-AS-BI-phosphohydrolase activities were present but esterase (C4), esteraselipase (C8), alkaline phosphatase, lipase (C14), valine arylamidase, cystine arylamidase, trypsin, α-chymotrypsin, α-galactosidase, β-galactosidase, β-glucuronidase, α-glucosidase, β-glucosidase, *N*-acetyl-β-glucosaminidase, α-mannosidase, and α-fucosidase activities are absent. The major fatty acids are iso-C _15__:__0_ and C _15__:__0_ 3-OH. MK-7 and MK-9 are present in minor amounts.

The type strain, Red111^T^ (= MCCC 1K04029^T^ = NBRC 114026^T^), was isolated from paddy soil of a field in Yamaguchi, Japan in April 2018. The genomic DNA G + C content of type strain is 61.0 mol%.

### Description of *Geomonas bemidjiensis* comb. nov.

*Geomonas bemidjiensis* (be.mid.ji.en’sis. N.L. fem. adj. *bemidjiensis* from Bemidji, MN, United States, where sediment samples were taken from which the type strain was isolated).

Basonym: *Geobacter bemidjiensis* Nevin et al., 2005.

Besides the characteristics that define the genus, the following characteristics are observed. Growth occurs at 15–37°C (optimum, 30°C) with the optimum pH, 7.0. With Fe(III)-NTA as electron acceptor, yeast powder, mannitol, glucose, tryptone, casamino acid, nicotinate, glycerol, isopropanol, serine, and proline can also be utilized as electron donor, but methanol, phenol, butanol, toluene, benzyl alcohol, arginine and benzaldehyde cannot be utilized as electron donor. With acetate as electron donor, malate, MnO_2_ and Fe(III) citrate can also be utilized as electron acceptor, but Fe(III) pyrophosphate, nitrate and sulfur cannot be utilized as electron acceptor. Leucine arylamidase, acid phosphatase, esterase (C4) and naphthol-AS-BI-phosphohydrolase activities were present, but esteraselipase (C8), alkaline phosphatase, lipase (C14), valine arylamidase, cystine arylamidase, trypsin, α-chymotrypsin, α-galactosidase, β-galactosidase, β-glucuronidase, α-glucosidase, β-glucosidase, *N*-acetyl-β-glucosaminidase, α-mannosidase, and α-fucosidase activities are absent. The major fatty acids are iso-C _15__:__0_, C_16__:__0_ and C _15__:__0_ 3-OH.

The type strain is Bem^T^ (= ATCC BAA-1014^T^ = DSM 16622^T^), isolated from subsurface sediments collected in Bemidji, MN, United States. The genomic DNA G + C content of type strain is 60.3 mol%.

### Description of *Geomonas bremensis* comb. nov.

*Geomonas bremensis* (bre.men’sis. N.L. fem. adj. *bremensis*, pertaining to Breme, in northern Germany, where samples for enrichment cultures were taken).

Basonym: *Geobacter bremensis* Straub and Buchholz-Cleven 2001.

As described by Straub and Buchholz-Cleven (2001), optimum growth occurs at 30–32°C with the optimum pH, 5.5–6.7. The majority of the cells are non-motile and tend to form aggregates. With ferrihydrite as electron acceptor, hydrogen, formate, acetate, propionate, butyrate, pyruvate, lactate, malate, succinate, fumarate, benzoate, ethanol, propanol and butanol can be utilized as electron donor. With acetate as electron donor, Fe(III), Mn(IV), sulfur, fumarate and malate can be utilized as electron acceptor. No vitamins required.

The type strain is Dfr1^T^ (= ATCC BAA-607^T^ = DSM 12179^T^), isolated from a freshwater ditch in Bremen, Germany. The genomic DNA G + C content of type strain is 60.0 mol%.

### Description of *Geomonas pelophila* comb. nov.

*Geomonas pelophila* (pe.lo’phi.la. Gr. masc. n. *pelos* mud; Gr. adj. *philos* loving; N.L. fem. adj. *pelophila*, mud-loving, as this species was isolated from freshwater mud).

Basonym: *Geobacter pelophilus* Straub and Buchholz-Cleven 2001.

As described by Straub and Buchholz-Cleven (2001), optimum growth occurs at 30–32°C with the optimum pH, 6.7–7.0. The majority of the cells are non-motile and tend to form aggregates. With ferrihydrite as electron acceptor, hydrogen, formate, acetate, propionate, pyruvate, malate, succinate, fumarate, ethanol and propanol can be utilized as electron donor. With acetate as electron donor, Fe(III), Mn(IV), sulfur, fumarate and malate can be utilized as electron acceptor. No vitamins required.

The type strain is Dfr2^T^ (= ATCC BAA-603^T^ = DSM 12255^T^), isolated from a freshwater ditch in Bremen, Germany. The genomic DNA G + C content of type strain is 60.9 mol%.

## Author Contributions

ZX performed most of the laboratory work and data analysis. ZX, YM, HI, and KS designed the experiment. HI and YS collected the samples from different fields. ZX and HI performed the enrichment and isolation work. NU took the TEM images. ZX and YM wrote the manuscript. ZX, YM, HI, and KS edited the manuscript.

## Conflict of Interest

The authors declare that the research was conducted in the absence of any commercial or financial relationships that could be construed as a potential conflict of interest.

## Footnotes

1 http://www.bacterio.net/geobacter.html

2 https://www.ezbiocloud.net/identify

3 www.ezbiocloud.net/tools/ubcg

4 http://jspecies.ribohost.com/jspeciesws/#analyse

5 https://ggdc.dsmz.de/ggdc.php

6 http://enve-omics.ce.gatech.edu/g

7 https://github.com/2015qyliang/POCP

8 http://textbookofbacteriology.net/growth_3.html

## Abbreviations

AAI: average amino acid identity
ANI: average nucleotide identity
DSMZ: Deutsche Sammlung von Mikroorganismen und Zellkulturen
GGDC: Genome-to-Genome-Distance Calculator
MFM: modified freshwater medium
MLSA: multilocus sequence analysis
POCP: Percentage of Conserved Proteins
RAPD: random amplified polymorphic DNA
rep-PCR: Repetitive sequence-based PCR
UBCG: up-to-date bacterial core gene.
