# Supplementary material for: Geomonas oryzae gen. nov., sp. nov., Geomonas edaphica sp. nov., Geomonas ferrireducens sp. nov., Geomonas terrae sp. nov., Four Ferric-Reducing Bacteria Isolated From Paddy Soil, and Reclassification of Three Species of the Genus Geobacter as Members of the Genus Geomonas gen. nov
Source: Front Microbiol. 2019 Sep 25;10:2201. doi: 10.3389/fmicb.2019.02201 (PMC6773877; doi:10.3389/fmicb.2019.02201)
Supplement: Supplementary file 1 [file Data_Sheet_1.PDF]

Supplementary Material

Geomonas oryzae **gen. nov., sp. nov.,** ***Geomonas edaphica*** **sp. nov.,** ***Geomonas ferrireducens*** **sp. nov.,** ***Geomonas terrae*** **sp. nov., four ferric-reducing bacteria isolated from paddy soil, and reclassification of three species of the genus** ***Geobacter*** **as members of the genus** ***Geomonas*** **gen. nov.**

Zhenxing Xu, Yoko Masuda, Hideomi Itoh, Natsumi Ushijima, Yutaka Shiratori, Keishi Senoo

Author for correspondence:

Dr. Yoko Masuda

Email: ygigico@gmail.com

frontiers

1. Supplementary Figures and Tables

1.1 Supplementary Tables

Supplementary Table 1. General genome features of four novel species: *Geomonas oryzae* S43^T, *Geomonas edaphica* Red53^T, *Geomonas ferrireducens* S62^T and *Geomonas terrae* Red111^T.

Features	**S43^T**	**Red53^T**	**S62^T**	**Red111^T**
Assembled contigs	18	17	16	8
Genome length (bp)	4,933,374	4,758,995	4,808,514	4,696,900
N50 length (bp)	578,332	1,148,776	1,386,104	1,057,769
Average G+C content (mol%)	61.2	60.5	60.7	61.0
Number of annotated ORFs	4170	4072	4098	3959
Number of RNAs	64	61	64	57
Depth of coverage (×)	236	757	647	299

Supplementary Table 2. The Average Amino Acid Identity (AAI) and the Percentage of Conserved Proteins (POCP) values between the four novel strains S43^T, Red53^T, S62^T, Red111^T and other known close species in the order *Desulfuromonadales.*

**Reference strains**[#]	**AAI value (%)**				**POCP value (%)**			
	S43^T	Red53^T	S62^T	Red111^T	S43^T	Red53^T	S62^T	Red111^T
Geomonas oryzae S43^T	100				100			
Geomonas edaphica Red53^T	93.7	100			88.0	100		
Geomonas ferrireducens S62^T	94.0	95.0	100		87.9	88.7	100	
Geomonas terrae Red111^T	94.3	96.1	95.4	100	88.1	89.4	88.1	100
Geobacter pelophilus Drf2^T	77.3	77.1	77.3	77.0	77.8	77.3	77.1	77.6
Geobacter bemidjiensis Bem^T	77.4	77.1	77.3	77.0	76.9	76.9	76.7	76.8
Geobacter bremensis R1	77.1	77.1	77.4	77.0	75.7	75.8	75.6	75.8
Geobacter uraniireducens Rf4^T	64.4	64.6	64.4	64.5	56.8	56.3	56.3	56.2
Geobacter daltonii FRC-32^T	63.4	63.1	63.3	62.7	58.6	58.4	58.4	57.5
Geobacter pickeringii G13^T	63.1	63.0	63.2	62.8	59.1	58.2	58.3	58.3
Geobacter metallireducens GS-15^T	62.2	62.0	62.4	61.9	57.8	56.5	57.3	56.7
Geobacter sulfurreducens PCA^T	62.2	62.0	62.2	61.8	58.7	58.7	58.1	58.5
Geobacter soli GSS01^T	62.0	62.0	62.3	61.8	58.2	57.4	57.7	57.6
Geobacter anodireducens SD-1^T	61.7	61.3	61.6	61.2	52.2	51.4	51.6	51.9
Geobacter toluenoxydans JCM 15764^T	60.8	60.6	60.6	60.4	53.0	52.1	52.7	52.1
Geobacter lovleyi SZ^T	57.9	57.9	57.8	57.7	49.5	49.8	49.4	49.7
Geobacter thiogenes ATCC BAA-34^T	57.8	57.5	57.5	57.5	48.3	48.3	48.0	48.1
Desulfuromonas soudanensis WTL^T	53.0	52.9	52.8	52.8	42.4	42.5	42.5	42.3
Desulfuromonas acetexigens DSM 1397^T	52.2	52.4	52.3	52.0	40.1	40.1	40.1	40.0
Desulfuromonas thiophila DSM 8987^T	49.6	49.7	49.6	49.4	33.8	33.7	33.2	33.2
Desulfuromonas acetoxidans DSM 684^T	48.6	48.6	48.6	48.5	32.9	32.8	32.8	32.8
Pelobacter seleniigenes DSM18267^T	49.3	49.4	49.3	49.0	34.8	34.8	34.8	34.0
Pelobacter acetylenicus DSM 3246^T	49.9	50.1	50.2	49.6	33.7	34.2	33.7	33.5
Pelobacter carbinolicus DSM 2380^T	49.9	50.0	49.9	49.5	35.1	35.2	35.1	34.4

[#]The whole annotated protein sequences of all reference strains were retrieved from NCBI database.

1.2 Supplementary Figures

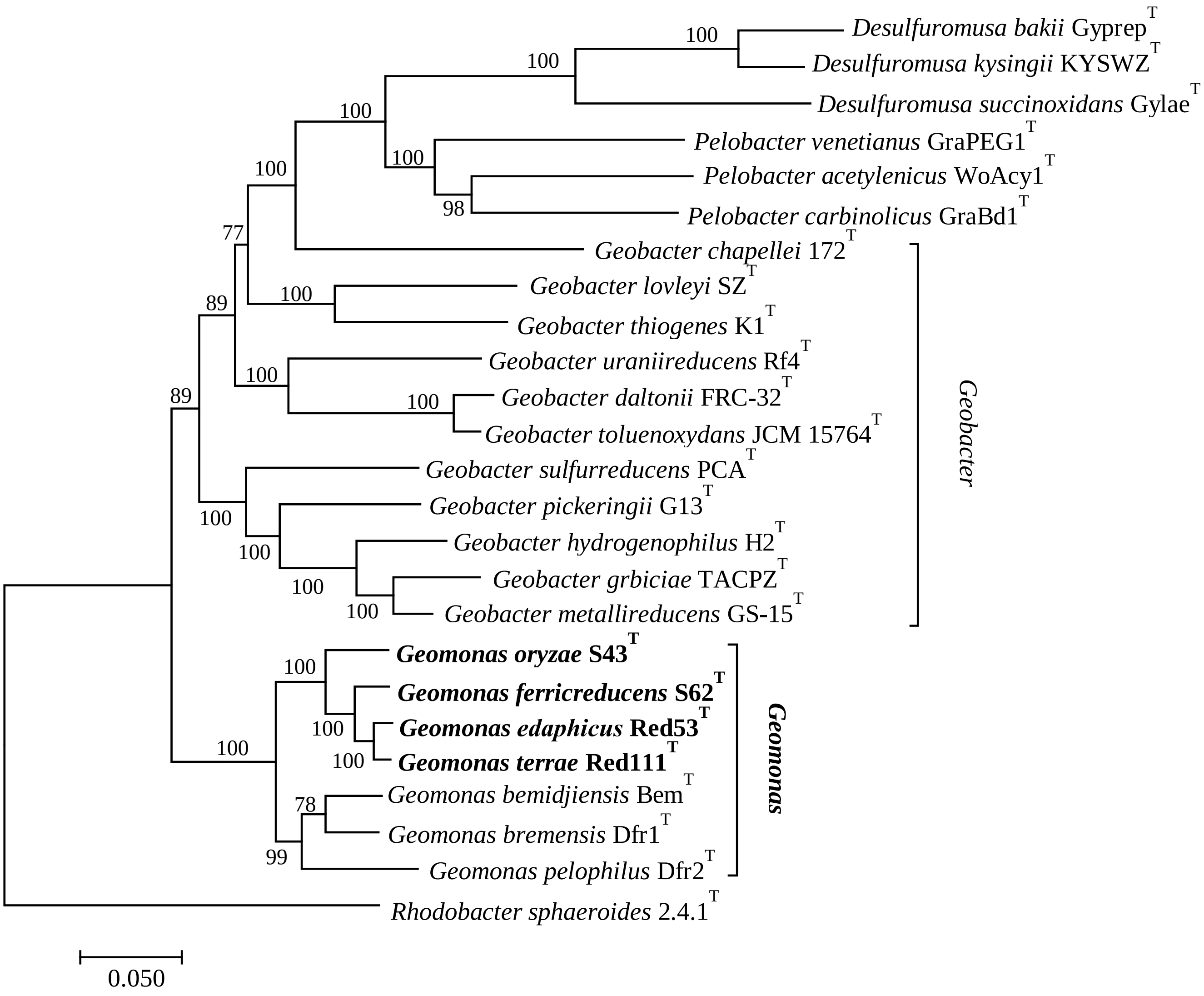

Supplementary Figure 1. Maximum-likelihood (ML) phylogenetic tree showing the position of strains S43^T, Red53^T, S62^T, Red111^T and representatives of some related taxa in the order *Desulfuromonadales* based on five concatenated housekeeping gene sequences: *rpoB* (1–525 bp), *recA* (526–1077 bp), *nifD* (1078–1809 bp), *gyrB* (1810–2670 bp) and *fusA* (2671–3124 bp). The tree was reconstructed using MEGA 7.0 with GTR+G+I model. Bootstrap values (expressed as percentages of 1, 000 replications) over 50% are shown at branching nodes. Bar, 0.05 substitutions per nucleotide position.

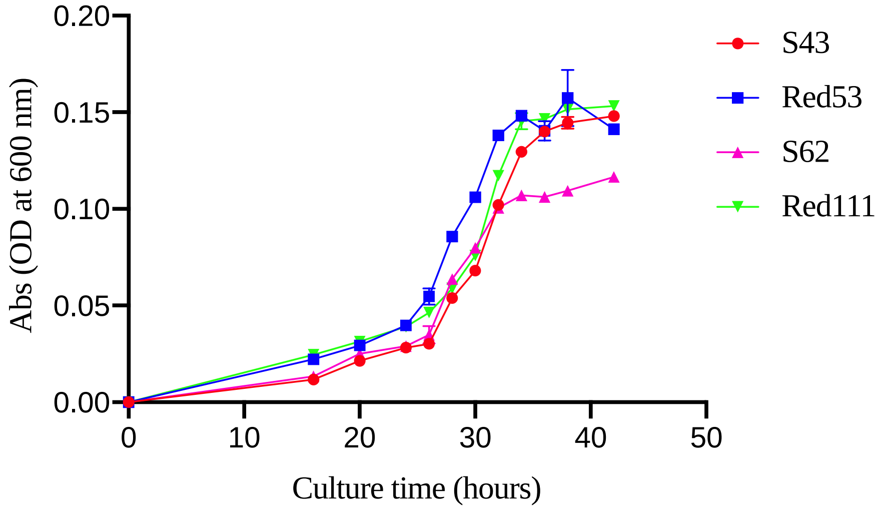

Supplementary Figure 2. The growth curves of the four isolated strains. Cells were grown on R2A liquid medium supplemented with 20 mM fumarate at 30 °C with 1/100 inoculation scale. The range of exponential phase is from 28th hour to 34th hour. Data were all presented as means ± standard deviations (SD) of triplicate. When not shown, error bars are smaller than the symbol size.

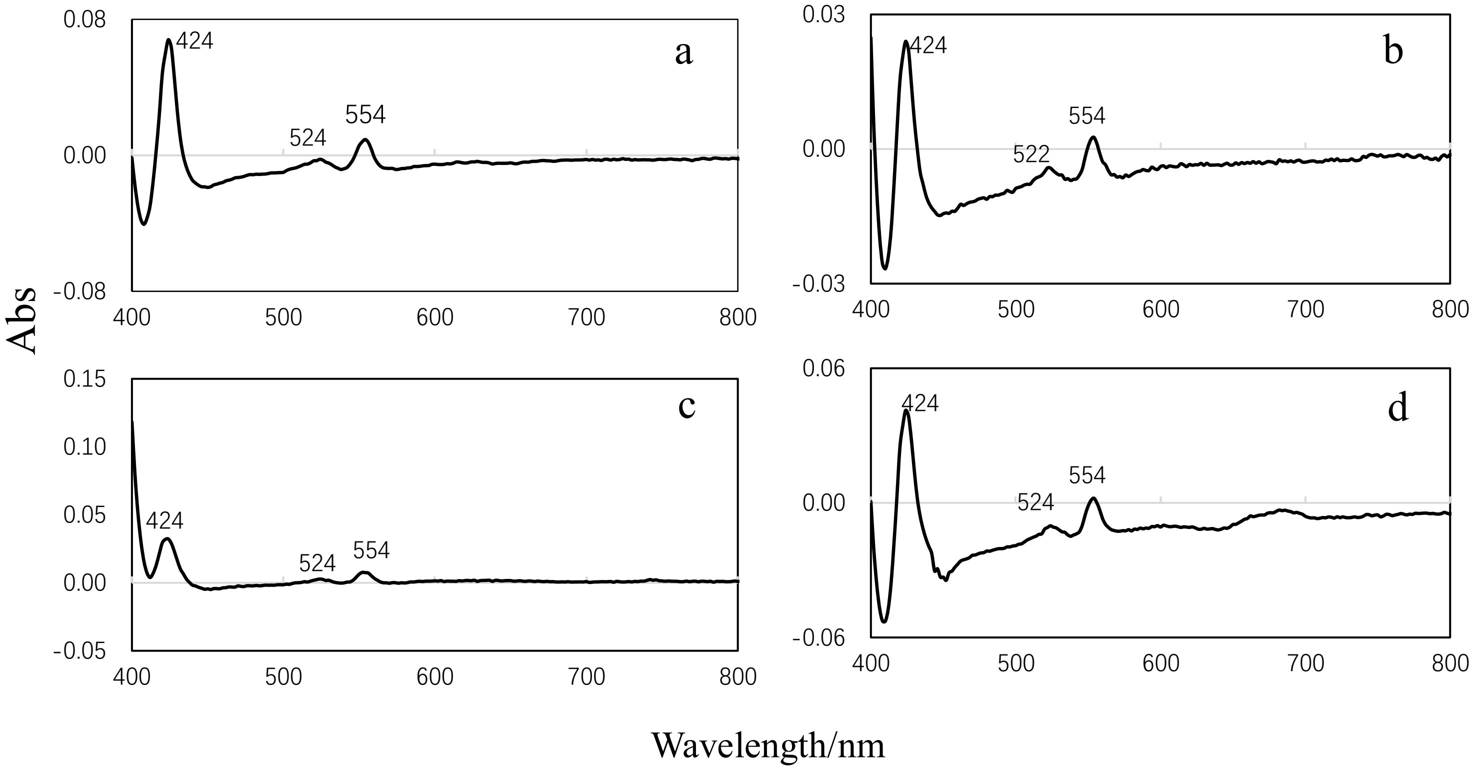

Supplementary Figure 3. Difference spectrum of whole cells for four novel species in the wavelength range of 400–800 nm. a, *Geomonas oryzae* S43^{T}; b, *Geomonas edaphica* Red53^{T}; c, *Geomonas ferrireducens* S62^{T}; d, *Geomonas terrae* Red111^{T}. The values shown in the figures were wavelength of every peak.

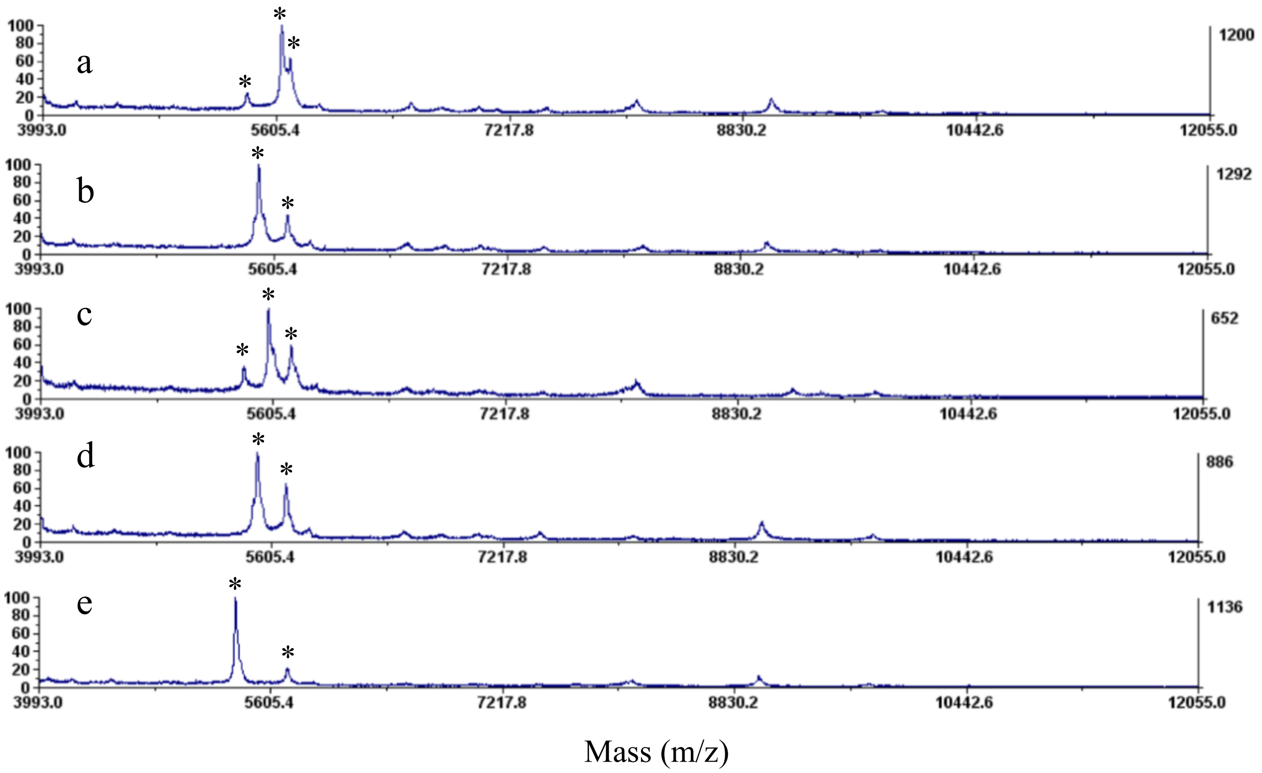

Supplementary Figure 4. MALDI-TOF mass spectra of five analysed strains in the range of m/z 4000 – 12000. a, *Geomonas oryzae* S43^T; b, *Geomonas edaphica* Red53^T; c, *Geomonas ferrireducens* S62^T; d, *Geomonas terrae* Red111^T; e, *Geobacter bemidjiensis* DSM 16622^T. The asterisks shown in the figures indicate the different peaks with other strains.
